# Predictors of Mortality Among Patients With Opioid Use Disorder: Insights From the Healthcare Cost and Utilization Project (HCUP) Nationwide Readmission Database

**DOI:** 10.7759/cureus.81405

**Published:** 2025-03-29

**Authors:** Naga Venkata Satish Babu Bodapati, Sandipkumar Patel, Rana Veer Samara Sihman Bharattej Rupavath, Omkar Reddy Polu, Balaiah Chamarthi, Chrishanti Anna Joseph

**Affiliations:** 1 Department of Psyhciatry, Sunshine Behavioral Health Services, Bakersfield, USA; 2 Department of Computer Engineering, Gujarat Technological University, Ahmedabad, IND; 3 Department of Business Administration, National Louis University, Tampa, USA; 4 Department of Technology and Innovation, City National Bank, Los Angeles, USA; 5 Department of Technology and Innovation, Info Services LLC, Livonia, USA; 6 Department of Anesthesiology and Perioperative Medicine, University of Pittsburgh, Pennsylvania, USA

**Keywords:** 30-day readmission, comorbidities and risk factors, healthcare cost and utilization project (hcup), mortality predictors, opioid use disorder (oud)

## Abstract

Background: Opioid use disorder (OUD) is associated with significantly increased mortality rates compared to the general population, driven by overdose risk, high-risk behaviors, and comorbid conditions. While opioid agonist treatment reduces mortality, identifying risk factors for death among individuals with OUD remains critical for improving outcomes.

Methods: A retrospective analysis of the 2020 National Readmission Database identified OUD admissions using International Classification of Diseases, 10th Revision, Clinical Modification codes. Patients over 18 years of age were included, and statistical analyses, including logistic regression, assessed 30-day readmission and mortality predictors. Data were analyzed using IBM Statistical Package for the Social Sciences Statistics for Windows, version 1.0.0.1327 (IBM Corp., Armonk, NY).

Results: Nonsurvivors were generally older (median age: 58 vs. 47 years) and had a higher prevalence of severe comorbidities, including cardiac arrest (24.1% vs. 0.4%, p < 0.001), respiratory failure (83.0% vs. 16.2%, p < 0.001), and acute kidney injury (61.9% vs. 16.8%, p < 0.001). Mortality was more common among patients with Medicare (44.4% vs. 31.7%) and in larger hospitals. Psychiatric conditions, such as depression and suicidal ideation, were more frequent in survivors, suggesting potential protective effects or earlier intervention. Multivariable analysis identified cardiac arrest (odds ratio, OR: 20.210, p < 0.001), respiratory failure (OR: 9.993, p < 0.001), and liver failure (OR: 4.298, p < 0.001) as the strongest mortality predictors, while female sex and psychiatric disorders were associated with lower mortality risk.

Conclusion: Mortality among patients with OUD is influenced by age, comorbidities, hospital characteristics, and healthcare disparities. Integrated care approaches that address both medical and psychiatric conditions are essential for improving survival outcomes. Future research should focus on targeted interventions to mitigate high-risk factors and enhance harm reduction strategies for this vulnerable population.

## Introduction

Opioid use disorder (OUD) is a complex and multifaceted condition related to significantly increased mortality rates compared to the general population [1]. Individuals with OUD face challenges such as heightened risks of overdose, diseases related to high-risk behaviors such as injecting drugs, and traumatic deaths. The standardized mortality ratio for individuals using nonmedical opioids is significantly elevated, with studies reporting standardized mortality ratios ranging from 10 to 24 times higher than the general population, indicating a substantial increase in mortality risk [2]. Despite these challenges, opioid agonist treatment has been shown to reduce all-cause and overdose-related mortality among individuals with OUD [3].

The implementation of opioid agonist treatment has been widely recognized as an effective strategy for managing OUD [4]. Studies have consistently demonstrated that being on opioid agonist treatment significantly decreases the risk of death compared to periods of treatment [5,6]. A study found that the off-versus-on opioid agonist treatment, all-cause mortality ratio was 2.31, showing the protective effect of continuous treatment engagement. Moreover, research shows that while on opioid agonist treatment, somatic causes account for a larger proportion of deaths than drug-induced causes [2].

Predictors of fatal outcomes in OUD are diverse and include both individual-level factors and broader societal influences. Individual characteristics such as increasing baseline age and a history of somatic hospital treatments have been linked to higher all-cause mortality risk [2]. Additionally, mental health comorbidities and coprescription of sedatives are known predictors of opioid overdose risk [7]. Social determinants like socioeconomic status also play a significant role in drug poisoning mortality rates across different populations [8].

Beyond individual health factors, environmental predictors such as release from prison or being on probation have been identified as significant risks for overdose among individuals with substance use disorders. These findings show the need for comprehensive approaches that address both medical vulnerabilities through interventions like methadone therapy or maintenance programs and social vulnerabilities by providing support services postrelease from incarceration [9].

This study of predictors of mortality for opioid disorder is crucial due to the alarming rise in opioid-related deaths. Factors such as higher doses, mental health comorbidities, and socioeconomic status are significant risk factors. Predictive modeling can guide targeted interventions by estimating overdose risk accurately. Understanding these factors is important for optimizing care strategies to reduce fatality rates among individuals with opioid addiction.

## Materials and methods

Study design

A retrospective analysis was conducted utilizing a publicly accessible database of all patients for 2020. We identified index admissions using diagnosis codes related to OUD. Because we employed a publicly accessible database, our work was exempt from Institutional Review Board requirements.

Data source

The National Readmission Database (NRD) provided data from January 1, 2020, to December 1, 2020. In the United States, NRD is the biggest all-patient, all-payer inpatient database. The NRD contains publicly accessible hospitalization data from nonfederal hospitals and is created and maintained by the Agency for Healthcare Research and Quality for the Healthcare Cost and Utilization Project (HCUP). The NRD 2020 includes discharge data from about 17 million unweighted discharges from 31 geographically separated states, representing 60.8% of all hospitalizations in the United States and 62.2% of all residents in the United States. It includes data at the hospital and patient levels. For each patient, the International Classification of Diseases, 10th Revision, Clinical Modification (ICD-10-CM/Procedure Coding System) is used to gather up to 40 discharge diagnoses and 25 procedures. A primary diagnosis is the cause for hospitalization, while a secondary diagnosis is any additional diagnostic that results in discharge. Hospitals are categorized by geographic region, teaching status, number of beds, ownership control, and urban/rural location. The NRD enables weighted analysis to determine all hospitalizations in the United States during a specific year.

Patients 18 years of age or older and whose primary diagnosis was an OUD, according to the ICD-10-CM codes, met the inclusion criteria. The exclusion criteria included the following: (1) individuals under 18 years old and (2) missing death data from the index admission.

Covariates

Age, gender, insurance status, median household income, loss of function, and discharge disposition were among the patient demographics gathered for the index admission. ICD-10-CM codes were also used to include comorbidities. We also collected hospital information, including bed size, ownership (private vs. government), designation (big, small, micropolitan, nonurban), and teaching status. ICD-10 procedure codes were used to identify the procedures performed throughout the hospital stay. The All Patients Refined Diagnosis Related Group (APRDRG) was used to determine the severity of the condition.

Statistical analysis

IBM Statistical Package for the Social Sciences Statistics for Windows, version 1.0.0.1327 (IBM Corp., Armonk, NY), was used for all statistical analyses. The Pearson chi-square test for categorical variables and the Mann-Whitney U-test for continuous variables with no mortality as the reference group were used to test for statistical differences in baseline participant characteristics, including age, gender, weekend vs. weekday admissions, household income, payer status, loss of function, and the likelihood of dying. Multivariable logistic regression was used to examine clinical factors for 30-day readmission. Additionally, each clinical variable's predictors of death were independently identified using multiple logistic regressions, and the results were displayed as odds ratios (OR) with 95% confidence intervals.

Patient and public involvement

This study uses the NRD, a publicly accessible all-payer inpatient medical readmission database in the United States derived from the Agency for Healthcare Research and Quality HCUP.

Data availability statement

The NRD is a sizable, publicly accessible all-payer inpatient care database in the United States, with information on over 17 million hospital visits [10].

## Results

The study analyzed patients admitted with opioid disorders, categorizing them by mortality status. Nonsurvivors were generally older (median age: 58 years) compared to survivors (47 years), with slightly more women in the mortality group. Elective admissions were significantly lower among nonsurvivors (2.2% vs. 9.2%, p < 0.001), while weekend admissions were marginally higher. Insurance status also differed, with Medicare being more common among nonsurvivors (44.4% vs. 31.7%, p < 0.001) and Medicaid coverage more frequent in survivors (42.7% vs. 33.6%, p < 0.001). The median hospitalization cost and length of stay were both significantly higher in nonsurvivors. Additionally, a greater proportion of nonsurvivors were classified in the extreme risk category of APRDRG, were more likely to be admitted to large hospitals, and had a higher likelihood of treatment at private nonprofit institutions (Table 1).

**Table 1:** Baseline characteristics during index admission for opioid disorder LOS: length of stay; IQR: interquartile range; APRDRG: all patients refined diagnosis-related group ^*^p < 0.05; ^**^p < 0.01 (statistically significant values) View larger

Characteristics	Mortality (n = 12,033; 1.8%)	No mortality (n = 644,030; 98.2%)	Overall (n = 656,063; 48.2%)	p value
Age (years), median (IQR)	47 (36-59)	58 (47-69)	46 (35-58)	-
Women	304,378; 47.3%	5,472; 45.5%	309,850; 47.2%	0.001^**^
Elective	59,364; 9.2%	264; 2.2%	59,628; 9.1%	0.001^**^
Weekend admission	157,568; 24.5%	3,168; 26.3%	160,736; 24.5%	0.001^**^
Insurance status				
Medicare	203,654; 31.7%	5,322; 44.4%	208,976; 31.9%	0.001^**^
Medicaid	274,688; 42.7%	4,029; 33.6%	278,717; 42.6%	0.001^**^
Private	89,823; 14%	1,428; 11.9%	91,251; 13.9%	0.001^**^
Self-pay	48,732; 7.6%	704; 5.9%	49,436; 7.5%	0.001^**^
No charge	7,037; 1.1%	53; 0.4%	7,090; 1.1%	0.001^**^
Other	19,002; 3%	457; 3.8%	19,459; 3%	0.001^**^
Cost of hospitalization in US$, median (IQR)	32,675 (24,189-42,897)	100,386.21 (75,210-128,530)	32,113 (24,000-42,500)	-
LOS, median (IQR)	4 (3-6)	6 (4-9)	4 (3-6)	-
Quartile of median household income				
0th-25th	231,672; 36.4%	4,219; 35.5%	235,891; 36.4%	0.045^*^
26th-50th^*^	176,953; 27.8%	3,286; 27.7%	180,239; 27.8%	0.045^*^
51st-75th	135,265; 21.2%	2,643; 22.3%	137,908; 21.3%	0.045^*^
76th-100th	92,755; 14.6%	1,727; 14.5%	94,482; 14.6%	0.045^*^
APRDRG, likelihood of dying				
Minor	126,171; 19.6%	48; 0.4%	126,219; 19.2%	0.001^**^
Moderate	262,426; 40.7%	258; 2.1%	262,684; 40.0%	0.001^**^
Major	170,606; 26.5%	1,531; 12.7%	172,137; 26.2%	0.001^**^
Extreme	84,668; 13.1%	10,195; 84.7%	94,863; 14.5%	0.001^**^
Hospital bed size				
Small	117,399; 18.2%	1,843; 15.3%	119,242; 18.2%	0.001^**^
Medium	180,192; 28%	3,364; 28%	183,556; 28.0%	0.001^**^
Large	346,438; 53.8%	6,826; 56.7%	353,264; 53.8%	0.001^**^
Control/ownership of hospital				
Government	78,211; 12.1%	1,437; 11.9%	79,648; 12.1%	0.001^**^
Private nonprofit	483,590; 75.1%	9,210; 76.5%	492,800; 75.1%	0.001^**^
Private for-profit	82,229; 12.8%	1,386; 11.5%	83,615; 12.7%	0.001^**^
Hospital designation				
Large metropolitan, ≥1 million residents	392,699; 61%	7,032; 58.4%	399,731; 60.9%	0.001^**^
Small metropolitan, ≤1 million residents	210,693; 32.7%	4,459; 37.1%	215,152; 32.8%	0.001^**^
Micropolitan	32,117; 5%	463; 3.8%	32,580; 5%	0.001^**^
Nonurban residual	8,521; 1.3%	79; 0.7%	8,600; 1.3%	0.001^**^
Hospital teaching status				
Metropolitan, nonteaching	120,923; 18.8%	2,317; 19.3%	123,240; 18.8%	0.001^**^
Metropolitan, teaching	482,468; 74.9%	9,174; 76.2%	491,642; 74.9%	0.001^**^
Nonmetropolitan	40,638; 6.3%	542; 4.5%	41,180; 6.3%	0.001^**^

Comorbidities played a critical role in mortality outcomes, with severe conditions significantly more prevalent among nonsurvivors. Cardiac arrest (24.1% vs. 0.4%, p < 0.001), respiratory failure (83.0% vs. 16.2%, p < 0.001), and shock (19.9% vs. 1.2%, p < 0.001) were notably higher in the mortality group. Renal complications, including acute kidney injury (61.9% vs. 16.8%, p < 0.001) and chronic kidney disease (22.1% vs. 11.1%, p < 0.001), were also significantly more frequent. Interestingly, psychiatric conditions such as depression and suicidal ideation were more common in survivors, suggesting a possible protective effect or earlier intervention. While survivors had higher rates of substance use disorders, pneumonia (36.8% vs. 9.9%, p < 0.001) and coronavirus disease 2019 (COVID-19) (11.6% vs. 2.0%, p < 0.001) were significantly more frequent among nonsurvivors (Table 2).

**Table 2:** Comorbidities and procedure related factors during index admission for opioid disorder AKI: acute kidney injury; COPD: chronic obstructive pulmonary disease; CKD: chronic kidney disease; PCI: percutaneous coronary intervention; GI: gastrointestinal; OCD: obsessive-compulsive disorder; COVID-19: coronavirus disease 2019 ^*^p < 0.01 (statistically significant values)

Comorbidities	Mortality (n = 12,033; 1.8%)	No mortality (n = 644,030; 98.2%)	Overall (n = 656,063; 47.1%)	p value
Cardiac arrest	2,417; 0.4%	2,895; 24.1%	5,312; 0.8%	0.001^*^
Heart failure	82,646; 12.8%	3,724; 31%	86,370; 13.2%	0.001^*^
Coronary atherosclerosis	80,434; 12.5%	2,505; 20.8%	82,939; 12.6%	0.001^*^
Shock	7,893; 1.2%	2,394; 19.9%	10,287; 1.6%	0.001^*^
Cardiac dysrhythmias	59,866; 9.3%	3,471; 28.8%	63,337; 9.7%	0.001^*^
AKI	107,937; 16.8%	7,451; 61.9%	115,388; 17.6%	0.001^*^
COPD	119,927; 18.6%	3,285; 27.3%	123,212; 18.8%	0.001^*^
CKD	71,574; 11.1%	2,661; 22.1%	74,235; 11.3%	0.001^*^
Respiratory failure	104,604; 16.2%	9,991; 83%	114,595; 17.5%	0.001^*^
Fluid and electrolyte disorders	232,011; 36%	9,626; 80%	241,637; 36.8%	0.001^*^
Cardiogenic shock	2,260; 0.4%	1,023; 8.5%	3,283; 0.5%	0.001^*^
Tobacco disorder	4,264; 0.7%	31; 0.3%	4,295; 0.7%	0.001^*^
Alcohol-related disorders	110,302; 17.1%	1,521; 12.6%	111,823; 17%	0.001^*^
Lipid disorders	122,929; 19.1%	2,632; 21.9%	125,561; 19.1%	0.001^*^
Hypertension	189,071; 29.4%	2,927; 24.3%	191,998; 29.3%	0.001^*^
Diabetes	125,007; 19.4%	2,861; 23.8%	127,868; 19.5%	0.007^*^
Obesity	88,499; 13.7%	1,680; 14%	90,179; 13.7%	0.487
GI bleed	14,783; 2.3%	1,114; 9.3%	15,897; 2.4%	0.001^*^
Liver failure	12,045; 1.9%	2,674; 22.2%	14,719; 2.2%	0.001^*^
Thyroid disorders	60,257; 9.4%	1,312; 10.9%	61,569; 9.4%	0.001^*^
Cancer	43,201; 6.7%	2,032; 16.9%	45,233; 6.9%	0.001^*^
Depression	179,278; 27.8%	1,989; 16.5%	181,267; 27.6%	0.001^*^
Pneumonia	63,639; 9.9%	4,432; 36.8%	68,071; 10.4%	0.001^*^
Pulmonary circulatory disorders	20,033; 3.1%	904; 7.5%	20,937; 3.2%	0.001^*^
Conduction disorder	14,117; 2.2%	641; 5.3%	14,758; 2.2%	0.001^*^
COVID-19	12,868; 2%	1,391; 11.6%	14,259; 2.2%	0.001^*^
Suicide attempt intentional self-harm	672; 0.1%	0; 0%	672; 0.1%	0.001^*^
Mental and substance use disorders	15,592; 2.4%	219; 1.8%	15,811; 2.4%	0.001^*^
Other specified substance-related disorders	38,692; 6%	412; 3.4%	39,104; 6%	0.001^*^
Hallucinogen-related disorders	286; 0%	3; 0%	289; 0%	0.313
Stimulant-related disorders	168,381; 26.1%	2,097; 17.4%	170,478; 26%	0.001^*^
Sedative-related disorders	53,567; 8.3%	501; 4.2%	54,068; 8.2%	0.001^*^
Cannabis-related disorders	87,802; 13.6%	714; 5.9%	88,516; 13.5%	0.001^*^
Neurodevelopmental disorders	25,015; 3.9%	135; 1.1%	25,150; 3.8%	0.001^*^
Miscellaneous mental and behavioral disorders conditions	2,115; 0.3%	19; 0.2%	2,134; 0.3%	0.001^*^
Suicidal ideation attempt intentional self-harm	70,107; 10.9%	214; 1.8%	70,321; 10.7%	0.001^*^
Somatic disorders	1,584; 0.2%	13; 0.1%	1,597; 0.2%	0.002^*^
Feeding and eating disorders	1,155; 0.2%	9; 0.1%	1,164; 0.2%	0.007^*^
Personality disorders	20,826; 3.2%	58; 0.5%	20884; 3.2%	0.001^*^
Disruptive impulse control and conduct disorders	2,384; 0.4%	9; 0.1%	2,393; 0.4%	0.001^*^
Trauma and stressor-related disorders	62,334; 9.7%	304; 2.5%	62,638; 9.5%	0.001^*^
OCD	3,532; 0.5%	23; 0.2%	3,555; 0.5%	0.001^*^
Anxiety and fear-related disorders	216,112; 33.6%	2241; 18.6%	218,353; 33.3%	0.001^*^
Other mood disorders	11,060; 1.7%	73; 0.6%	11,133; 1.7%	0.001^*^
Bipolar disorders	78,540; 12.2%	611; 5.1%	79,151; 12.1%	0.001^*^
Schizophrenia	43,228; 6.7%	341; 2.8%	43,569; 6.6%	0.001^*^

**Figure 1:**
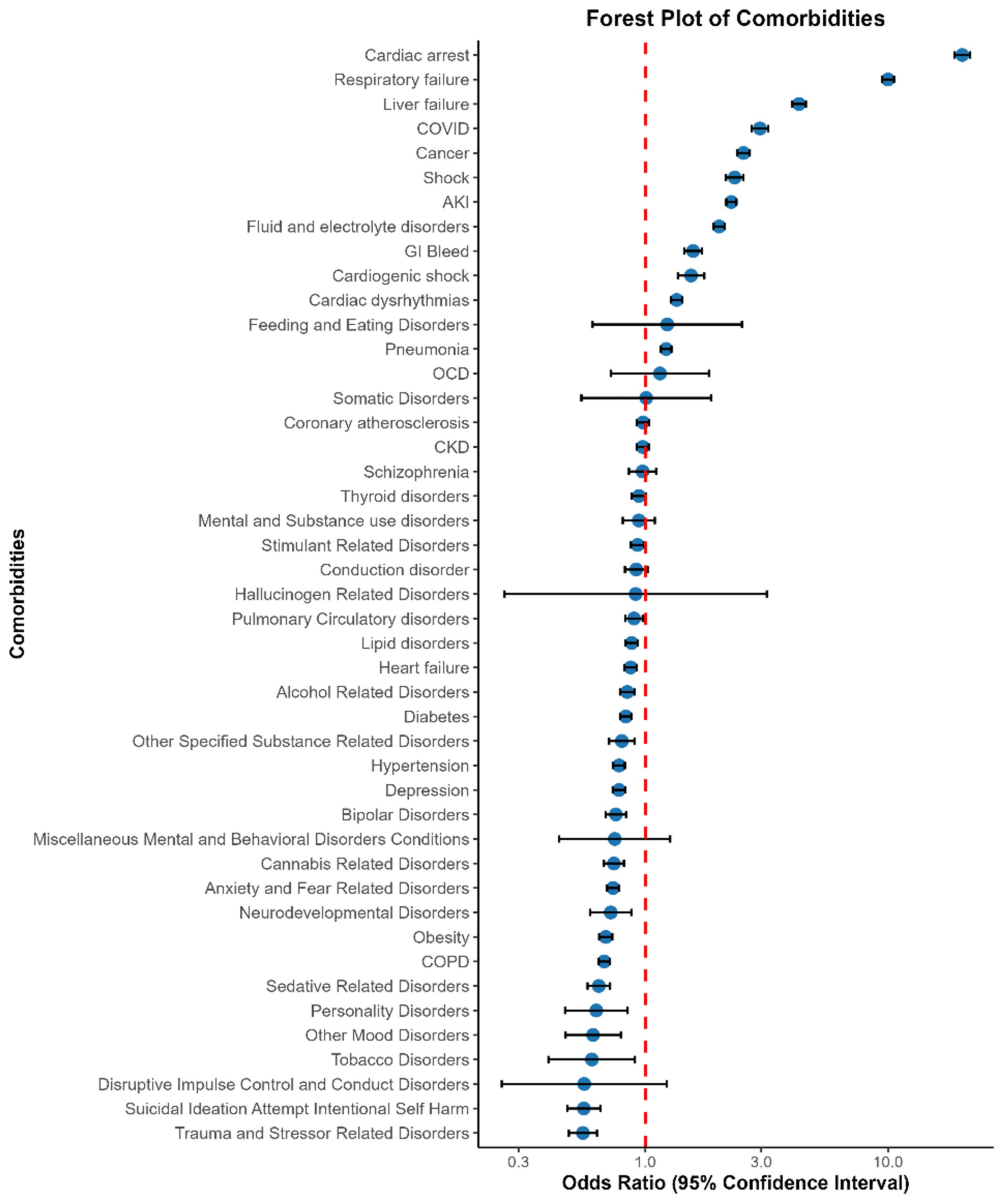
Forest plot of comorbidities associated with mortality in opioid use disorder AKI: acute kidney injury; GI: gastrointestinal; CKD: chronic kidney disease; OCD: obsessive-compulsive disorder; COPD: chronic obstructive pulmonary disease

Multivariable analysis identified key predictors of mortality, with younger age groups (18-44 and 45-64 years) having significantly lower odds of death compared to those aged ≥75 years. Female patients had a reduced mortality risk (OR: 0.904, p < 0.001), and Medicaid patients had higher mortality odds compared to Medicare and privately insured patients. Hospital characteristics also influenced outcomes, with larger hospitals associated with greater mortality risk (OR: 1.136, p < 0.001). Among comorbidities, cardiac arrest (OR: 20.210, p < 0.001), respiratory failure (OR: 9.993, p < 0.001), and liver failure (OR: 4.298, p < 0.001) were the strongest predictors of death. In contrast, mental health conditions such as depression and bipolar disorder were linked to lower mortality risk, while suicidal ideation also had an inverse association with mortality (OR: 0.558, p < 0.001) (Table 3, Figure 1).

**Table 3:** Multivariable logistic regression analysis for predicting mortality OR: odds ratio; CI: confidence interval; AKI: acute kidney injury; COPD: chronic obstructive pulmonary disease; CKD: chronic kidney disease; GI: gastrointestinal; COVID-19: coronavirus disease 2019; OCD: obsessive-compulsive disorder ^*^p < 0.05, ^**^p < 0.01 (statistically significant values)

Variable	OR (95% CI)	p value
Age (years)		
≥75	1 (reference)	-
18-44	0.379 (0.343-0.418)	0.001^**^
45-64	0.556 (0.513-0.603)	0.001^**^
65-74	0.711 (0.658-0.769)	0.001^**^
Sex		
Male	1 (reference)	-
Female	0.904 (0.864-0.946)	0.001^**^
Insurance status		
Medicaid	1 (reference)	-
Medicare	0.890 (0.834-0.949)	0.001^**^
Private insurance	0.838 (0.777-0.903)	0.185
Self-pay	1.022 (0.925-1.129)	0.666
No charge	0.590 (0.426-0.817)	0.002^**^
Other	1.418 (1.257-1.601)	0.001^**^
Hospital bed size		
Small	1 (reference)	-
Medium	1.084 (1.013-1.160)	0.020^*^
Large	1.136 (1.067-1.208)	0.001^**^
Control/ownership of hospital		
Government	1 (reference)	-
Private nonprofit	1.001 (0.936-1.071)	0.969
Private for profit	0.957 (0.874-1.048)	0.348
Hospital designation		
Large metropolitan, ≥1 million residents	1.166 (1.113-1.221)	0.001^**^
Small metropolitan, ≤1 million residents	0.970 (0.870-1.082)	0.589
Micropolitan	0.871 (0.676-1.123)	0.287
Comorbidities		
Cardiac arrest	20.210 (18.774-21.756)	0.001^**^
Heart failure	0.870 (0.821-0.920)	0.001^**^
Coronary atherosclerosis	0.978 (0.923-1.037)	0.453
Shock	2.335 (2.149-2.537)	0.001^**^
Cardiac dysrhythmias	1.346 (1.276-1.420)	0.001^**^
AKI	2.259 (2.155-2.368)	0.001^**^
COPD	0.677 (0.643-0.714)	0.001^**^
CKD	0.975 (0.920-1.033)	0.394
Respiratory failure	9.993 (9.445-10.571)	0.001^**^
Fluid and electrolyte disorders	2.013 (1.910-2.122)	0.001^**^
Cardiogenic shock	1.543 (1.362-1.749)	0.001^**^
Tobacco disorders	0.602 (0.399-0.906)	0.015^*^
Alcohol-related disorders	0.842 (0.787-0.902)	0.001^**^
Lipid disorders	0.878 (0.829-0.929)	0.001^**^
Hypertension	0.779 (0.737-0.823)	0.001^**^
Diabetes	0.831 (0.787-0.877)	0.016^*^
Obesity	0.687 (0.645-0.731)	0.001^**^
GI bleed	1.573 (1.447-1.711)	0.001^**^
Liver failure	4.298 (4.029-4.586)	0.001^**^
Thyroid disorders	0.941 (0.878-1.008)	0.083
Cancer	2.537 (2.392-2.691)	0.001^**^
Depression	0.779 (0.734-0.826)	0.001^**^
Pneumonia	1.218 (1.158-1.281)	0.001^**^
Pulmonary circulatory disorders	0.898 (0.826-0.976)	0.001^**^
Conduction disorder	0.917 (0.824-1.021)	0.113
COVID-19	2.966 (2.747-3.203)	0.001^**^
Mental and substance use disorders	0.940 (0.807-1.094)	0.423
Other specified substance-related disorders	0.800 (0.709-0.902)	0.001^**^
Hallucinogen-related disorders	0.912 (0.263-3.166)	0.885
Stimulant-related disorders	0.929 (0.872-0.990)	0.022^*^
Sedative-related disorders	0.643 (0.578-0.715)	0.001^**^
Cannabis-related disorders	0.742 (0.675-0.816)	0.001^**^
Neurodevelopmental disorders	0.720 (0.592-0.876)	0.001^**^
Miscellaneous mental and behavioral disorders conditions	0.748 (0.442-1.265)	0.279
Suicidal ideation attempt intentional self-harm	0.558 (0.477-0.653)	0.001^**^
Somatic disorders	1.008 (0.545-1.865)	0.980
Feeding and eating disorders	1.231 (0.606-2.500)	0.566
Personality disorders	0.628 (0.467-0.845)	0.002^**^
Disruptive impulse control and conduct disorders	0.560 (0.256-1.226)	0.147
Trauma and stressor-related disorders	0.553 (0.484-0.632)	0.001^**^
OCD	1.149 (0.722-1.829)	0.557
Anxiety and fear-related disorders	0.736 (0.695-0.778)	0.001^**^
Other mood disorders	0.609 (0.469-0.793)	0.001^**^
Bipolar disorders	0.756 (0.686-0.833)	0.001^**^
Schizophrenia	0.974 (0.856-1.108)	0.689

## Discussion

This study highlights the multifaceted interplay of demographic, socioeconomic, clinical, and institutional factors influencing mortality among patients with OUD. The findings underscore significant disparities in healthcare access, comorbidity burden, and hospital-related factors contributing to differential survival outcomes in this vulnerable population.

Our study demonstrates that older adults with OUD are at a substantially higher risk of mortality, with nonsurvivors being significantly older than survivors (median age: 58 vs. 47 years). This finding is consistent with previous research, which suggests that aging individuals with OUD face unique challenges, including increased physiological vulnerability to opioid toxicity, a higher burden of multimorbidity, and decreased engagement in harm reduction services [11]. Moreover, Medicare recipients were more prevalent among nonsurvivors, further reinforcing that older adults with OUD may have reduced access to timely and specialized addiction treatment. Studies have shown that older adults with OUD are less likely to be prescribed opioid agonist therapy (OAT) and more likely to have unmanaged chronic pain, which can lead to inappropriate opioid prescribing and a higher risk of adverse outcomes [12].

The lower rate of elective admissions and a higher rate of weekend admissions in nonsurvivors suggest that these patients may delay seeking care until their condition becomes critical, necessitating emergency intervention. This aligns with previous studies indicating that patients with OUD often experience barriers to preventive care, leading to more severe acute presentations upon hospital admission [13]. Furthermore, the significant disparity in insurance status between survivors and nonsurvivors, where Medicaid was more prevalent among survivors, points to the role of healthcare accessibility in reducing mortality. Medicaid expansion has been associated with improved access to OAT and harm reduction programs, which are critical in preventing opioid-related deaths [14].

Comorbidities were among the strongest determinants of mortality in our study, with nonsurvivors exhibiting a significantly higher prevalence of conditions such as cardiac arrest, respiratory failure, shock, and renal complications. These findings align with previous research demonstrating that OUD is frequently accompanied by serious medical conditions that increase the risk of in-hospital mortality [12]. Respiratory failure was particularly prevalent in the nonsurvivor group, consistent with established knowledge that opioid overdose-induced respiratory depression is the leading cause of opioid-related deaths [15]. Prior studies have also highlighted that opioid use impairs immune function, increasing susceptibility to severe respiratory infections such as pneumonia and COVID-19, which were significantly more frequent among nonsurvivors in our cohort [16].

Interestingly, psychiatric conditions such as depression and suicidal ideation were more common in survivors, suggesting that patients receiving mental health care may have better engagement with healthcare services, ultimately reducing their mortality risk. This is consistent with studies showing that individuals with co-occurring psychiatric disorders are more likely to seek medical care and be enrolled in treatment programs, which may serve as a protective factor [3]. However, it is also possible that psychiatric diagnoses were underreported in nonsurvivors due to lower healthcare utilization or stigma surrounding mental health conditions in patients with substance use disorders.

Our multivariable analysis identified older age as one of the strongest predictors of mortality, corroborating previous studies that have demonstrated an exponential increase in opioid-related deaths among aging populations [17]. The protective effect of female sex observed in our study may be attributed to biological and behavioral differences, including variations in opioid metabolism, hormonal influences, and healthcare-seeking behaviors. Prior research has similarly found that opioid-related mortality rates are consistently higher in men than in women, potentially due to differences in risk-taking behavior and opioid prescription patterns [18].

The finding that Medicaid patients had higher mortality odds compared to those with Medicare or private insurance highlights persistent disparities in healthcare quality and access. While Medicaid covers OUD treatment, variations in state-level policies and provider availability may contribute to inconsistent access to evidence-based interventions such as buprenorphine and methadone maintenance therapy. Similar studies have reported that Medicaid recipients with OUD often face longer wait times for treatment and higher rates of discontinuation, both of which increase mortality risk [19].

Sex differences in psychiatric comorbidities may further influence mortality in OUD. Women with OUD have higher rates of depression, anxiety, and posttraumatic stress disorder, which may enhance healthcare engagement and access to integrated treatment, contributing to lower mortality [20]. Conversely, men may face greater stigma in seeking mental health care, leading to untreated psychiatric conditions and worse outcomes [21]. The higher prevalence of psychiatric diagnoses in survivors suggests that mental health care access may play a protective role, though underreporting in nonsurvivors remains a concern.

Additionally, our study found that larger hospitals were associated with greater mortality risk, a finding that may reflect the complexity of care in these institutions. While large hospitals often have more resources, they may also have higher patient loads, which can lead to longer wait times, increased risk of medical errors, and challenges in providing individualized addiction treatment. Previous research has suggested that patients with OUD treated in large, urban hospitals may experience fragmented care due to poor coordination between emergency departments, addiction specialists, and primary care providers [4].

Cardiac arrest, respiratory failure, and liver failure were among the strongest predictors of mortality in our study, emphasizing the importance of addressing these critical complications in patients with OUD. These findings align with prior studies demonstrating that opioid use is associated with progressive organ damage, including cardiopulmonary and hepatic dysfunction, which significantly increases the risk of death [22]. The strong association between respiratory failure and mortality highlights the need for widespread availability of naloxone and improved hospital-based interventions for opioid-induced respiratory depression.

Study limitations

This study has several limitations. First, the retrospective design and reliance on administrative data may introduce coding errors or misclassification biases. Second, the dataset does not capture nonhospitalized opioid-related deaths, potentially underestimating mortality rates. Third, factors such as the type and dosage of opioids used, adherence to OAT, and social determinants of health were not available in the dataset, limiting the ability to explore their impact on mortality. Additionally, while the study identifies associations, causal relationships cannot be inferred. Future research should incorporate prospective designs and more granular patient-level data to better understand the mechanisms driving mortality in OUD populations.

## Conclusions

In conclusion, this study provides valuable insights into the factors contributing to mortality among patients with OUD. The findings highlight the need for comprehensive, integrated care that addresses medical and psychiatric comorbidities, reduces disparities in healthcare access, and promotes harm reduction strategies. Future research should focus on identifying specific interventions to mitigate the identified risk factors and improve outcomes for this vulnerable population
